# Voriconazole-Induced Acute Liver Injury: A Case Report

**DOI:** 10.7759/cureus.20115

**Published:** 2021-12-02

**Authors:** Ramakanth Pata, Tsering Dolkar, Meet Patel, Nway Nway

**Affiliations:** 1 Pulmonary and Critical Care Medicine, One Brooklyn Health, New York, USA; 2 Pulmonary and Critical Care Medicine, University of Cincinnati Medical Center, Cincinnati, USA; 3 Internal Medicine, Interfaith Medical Center, Brooklyn, USA

**Keywords:** riociguat, macitentan, pulmonary hypertension, voriconazole, drug-induced liver injury

## Abstract

Drug-induced liver injury (DILI) is the most common cause of acute liver failure in the United States. Clinical presentation of drug-induced liver injury may vary from asymptomatic or subtle symptoms to encephalopathy with serious morbidity. Early discontinuation of the offending agent is important to prevent clinical deterioration. Occasionally, despite discontinuation, there may be worsening of liver failure with grim prognosis as we present in this case report. Here, we report a case of a 61-year-old lady with a past medical history of sarcoidosis, stage IV and severe pulmonary hypertension initially admitted for the management of COVID pneumonia. Her hospitalization was complicated by fungemia with Aspergillus for which voriconazole was initiated, and two weeks into the course, acute liver injury diagnosed was most probably related to voriconazole. Despite discontinuation, her condition deteriorated, eventually culminating in mortality.

## Introduction

Drug-induced liver injury (DILI) is considered to be the most common cause of acute liver failure in the United States, accounting for 10% of all cases of acute hepatitis. There have been many medications both prescription and over-the-counter drugs including the herbal ones that have been implicated in DILI. Risk factors that have been associated with DILI are female gender, alcohol use, and malnutrition. LiverTox is one of the most cited online databases to search for an offending medication. The clinical presentation of DILI may be subtle and asymptomatic or florid with symptoms and signs consistent with acute liver failure. Diagnosis is confirmed with biochemical evidence of liver injury and the patterns of liver enzyme elevation. The patterns of drug-induced liver injury may be classified predominantly as either hepatocellular (alanine aminotransferase (ALT) > 3 upper limit of normal (ULN)) or cholestatic (alkaline phosphatase (ALP) > 2 ULN) or mixed pattern with a combination of the two [1]. Diagnosis is also made in the presence of an offending agent with any elevation of ALT or ALP if accompanied by total bilirubin > 2 ULN. Histological correlates include hepatitis, cholestasis, and steatosis. Treatment consists of supportive care and discontinuation of the drug responsible for DILI. There are no specific therapies except for N-acetylcysteine for acetaminophen toxicity and L-carnitine for valproate overdose. As most cases of drug-induced liver injury resolve after discontinuation of the offending agent and with supportive management, it is crucial to recognize the liver injury at an early stage. Occasionally, despite discontinuation, clinical deterioration may ensue leading to morbidity and mortality. Prognostically, most patients exhibit complete recovery except in a rare patient with a cholestatic pattern that persists up to one year or may lead to progressive cholestasis known as vanishing bile duct syndrome. Poor prognosis factors typically include elevated serum creatinine and preexisting liver injury. Hy's law, although rare with an incidence of 0.7-1.3 per 100,000, has traditionally been quoted as a poor prognostic factor and has been associated with 14%-80% mortality. Hy's law is defined as the development of jaundice with bilirubin > 2 ULN and ALT > 3 ULN [2]. Overall, chronic disease may occur in up to 6% even if the offending drug is withdrawn. Antibiotics and nonsteroidal anti-inflammatory drugs (NSAIDs) remain the most common causes of DILI [3].

## Case presentation

**Figure 1:**
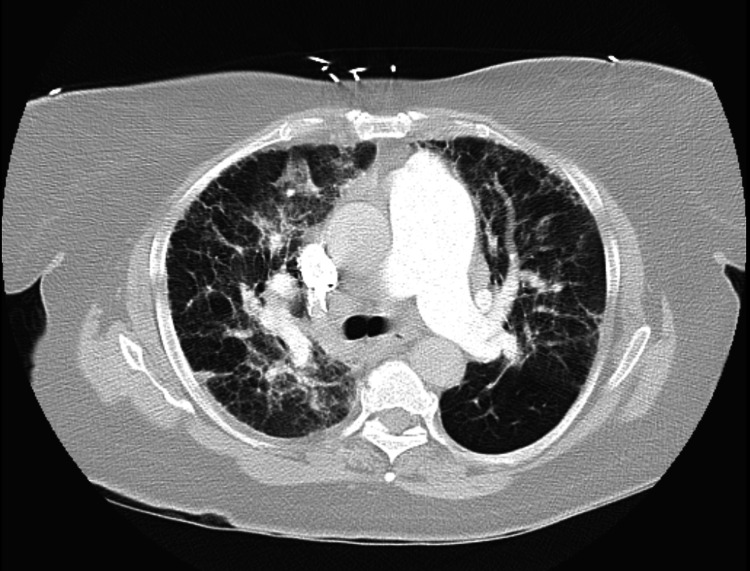
CT chest axial view lung window showed diffuse bilateral multifocal ground-glass opacities with prominent interlobular septa in the lower lobes.

**Figure 2:**
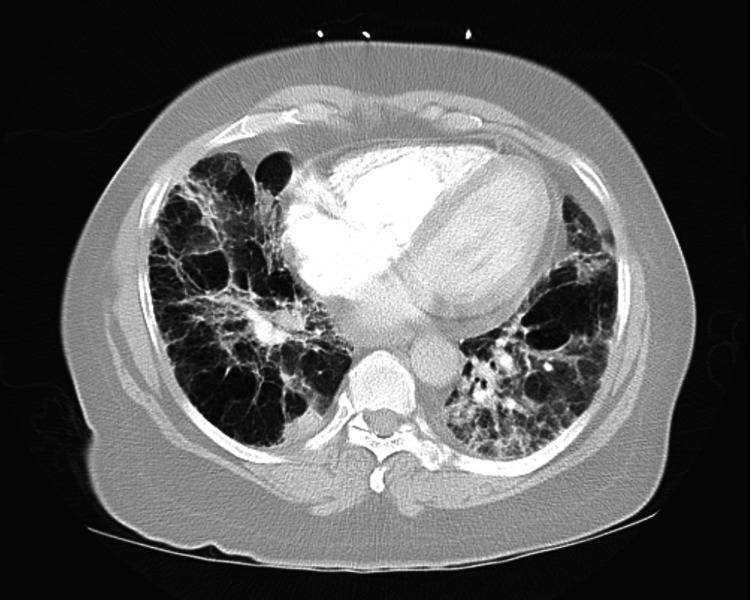
Chest CT axial section at the level of pulmonary trunk showing bilateral scattered patchy infiltrates suggestive of multifocal pneumonia superimposed on centrilobular emphysema.

A 61-year-old lady with a past medical history of sarcoidosis, stage IV with interstitial lung disease (on prednisone 20 mg daily), severe pulmonary hypertension (on home medications: macitentan and riociguat), presented to the emergency department for progressively worsening cough, shortness of breath (SOB), and low-grade fever from the past four days. The patient also endorsed generalized body fatigue and decreased appetite. Upon presentation, temperature was 98.1°F, pulse was 87/min, respiratory rate (RR) was 20/min, and blood pressure (BP) was 101/53 mm Hg. Patient was saturating at 84% on room air and > 95% with 2 L nasal cannula. The patient was conscious, alert, and oriented to time, place, and person. Physical examination was significant for bilateral crepitations more in the right lung with associated decreased breath sounds. Lab workup was notable for increased lactate dehydrogenase (LDH) (348), creatinine kinase (CK) (393), interleukin (IL)-6 (44.4), and D-dimer (597). Chest X-ray was consistent with chronic interstitial opacities with fibrosis. CT pulmonary angiogram was done, to rule out pulmonary embolism, which revealed diffuse asymmetric bilateral multifocal airspace disease with reticulations (Figures 1, 2).

COVID polymerase chain reaction (PCR) test was positive. She was managed for COVID-19 pneumonia with dexamethasone, empirical antibiotics, and oxygen supplementation as required that included a high-flow nasal cannula on day 5 and did not require intubation during this early phase. Macitentan and riociguat were continued as the patient had a history of severe pulmonary hypertension with mean pulmonary arterial pressure (mPAP) of 90 mm Hg (diagnosed via right heart catheterization in October 2017). The patient was transferred to floor service and was not discharged as she still required oxygen at 6-7 L/min. On day 27, the condition worsened with febrile spikes accompanied by increasing requirements of oxygen needing an ICU upgrade. Sputum culture, blood culture, and procalcitonin were sent and returned negative. Further diagnostic workup included fungal cultures, serum and sputum galactomannan, and beta-D glucan. Broad-spectrum antimicrobials were started that included caspofungin, vancomycin, and meropenem. Sputum Aspergillus galactomannan antigen level was elevated above normal with a value 0.62, and fungal blood culture was positive for acutely branching septate hyphae consistent with Aspergillosis. Caspofungin was switched to voriconazole to cover Aspergillosis. The patient was started on voriconazole 300 mg twice a day (BID) on day 39 of admission. It was not started immediately after the fungal result as there was an interaction with macitentan (category X interaction) and riociguat (category C), which mandated complete discontinuation of macitentan and a safe period of drug wash out to initiate voriconazole. Riociguat was continued throughout her stay in the hospital. Echocardiography was obtained which revealed normal left ventricular systolic dysfunction. Echo findings revealed markedly dilated and hypokinetic right ventricle, PASP of 90 mm Hg, and dilated right atrium with severe tricuspid regurgitation. This decision was made after an intensive multidisciplinary discussion with intensivist, cardiologist, pulmonary hypertension center, and an infectious disease (ID) specialist. After 72 hours, there was a tremendous improvement in her respiratory status and later transferred to the Medical Surgical Unit requiring only 2-3 L of oxygen. As macitentan was discontinued and echo revealed elevated pulmonary pressures, IV Remodulin (treprostinil) was initiated to offload the right ventricle with gradual titration of the dose. As the worsening of hypoxia was temporally related to uptitration, which was documented on three occasions, treprostinil was later discontinued. The plan was to continue voriconazole at least for two weeks for maintenance and she remained stable requiring only 2 L/min of oxygen.

On day 52, the patient reported fullness in the abdomen and fluctuating mental status that required readmission to the ICU. A comprehensive metabolic panel revealed elevated transaminases and serum creatinine (Table 1). Considering that voriconazole could be the reason for acute hepatic failure, a decision was made to discontinue voriconazole, and supportive management including N-acetylcysteine was administered, after a shared decision with the family. 

**Table 1:** Trend of liver enzymes after starting voriconazole treatment. Voriconazole was stopped on day 22 of voriconazole initiation.

Time Since Voriconazole Treatment	ALT (Alanine Aminotransferase)	AST (Aspartate Aminotransferase)	INR (International Normalized Ratio)
Day 1	17	30	1.03
Day 20	36	37	3.33
Day 21	88	54	4.61
Day 22	865	330	5.96
Day 23	1,303	495	5.53
Day 24	1,169	552	6.28
Day 25	1,692	766	2.66
Day 26	>3,600	1,173	2.75
Day 27	3,390	1,364	3.78
Day 28	1,812	1,178	4.10
Day 29	979	813	3.53

During the ICU stay, the transaminases progressively increased, international normalized ratio (INR) worsened, aspartate aminotransferase (AST)/ALT levels increased to > 3,600/1,364, ALP level increased to 170 U/L, INR increased to 6.28, and oxygen requirement increased from 8 L on an oxygen mask to high-flow nasal cannula 60 L and fractional inspired oxygen (FiO_2_) 100% and eventually requiring intubation. On day 64, the hemodynamic status worsened requiring multiple vasopressors (norepinephrine, vasopressin, and dobutamine) and bedside echo was suggestive of hyperdynamic circulation. Lung transplant center was consulted, and the patient was not a suitable candidate for transplant due to severe pulmonary hypertension. Unfortunately, the patient demised on day 67.

## Discussion

Acute liver failure refers to the development of severe acute liver injury with encephalopathy and impaired synthetic function (INR of ≥ 1.5) in a patient without cirrhosis or preexisting liver disease [4]. Acute liver failure is a rare and severe consequence of abrupt hepatocyte injury and can evolve over days or weeks to a lethal outcome. A variety of insults to liver cells result in a consistent pattern of rapid-onset elevation of aminotransferases, altered mentation, and disturbed coagulation. In the United States, the US Acute Liver Failure Study Group collected data on 1,147 cases of acute liver failure from 23 sites between 1998 and 2007. The most common causes of acute liver failure were acetaminophen overdose (46%), indeterminate (14%), idiosyncratic drug reactions (12%), hepatitis B virus (7%), and hepatitis A virus (3%) [5]. The absence of existing liver disease distinguishes acute liver failure from decompensated cirrhosis or acute-on-chronic liver failure [4]. Acute liver failure has been studied widely in randomized controlled trials because of its rarity. Clinical case reports of drug-induced acute liver failure become valuable information for reflection for prevention and cautiousness in clinical practice. 

The patient described in our case has been on chronic steroids for sarcoidosis and was being treated for pulmonary hypertension. These comorbidities and COVID infection may have put her at increased risk of Aspergillosis, which is best treated by voriconazole. 

Voriconazole is a triazole antifungal agent used primarily in the treatment or prevention of Aspergillosis and candidal infections. Risk factors for Aspergillosis are usually immunocompromised states that include hematopoietic stem cell transplant (HSCT), solid transplant, and chronic steroid therapy. Voriconazole therapy is associated with transient, asymptomatic serum aminotransferase elevations and is a known cause of clinically apparent acute drug-induced liver injury. Several other classes of antifungal agents like polyenes (amphotericin B) and echinocandins (micafungin) can also rarely cause liver injury leading to severe transaminitis/hyperbilirubinemia and sometimes liver failure as well [6]. Transient elevations in serum aminotransferase levels occur in 11% to 19% of patients on voriconazole. These elevations are usually asymptomatic and self-limited, but approximately 1% of patients require discontinuation of voriconazole because of elevated liver enzymes. Clinically apparent hepatotoxicity is uncommon with voriconazole and the reason behind it is unknown. It could be related to alteration in sterol synthesis by voriconazole. Also, voriconazole is metabolized by different CYP450 enzymes which may result in multiple drug-drug interactions. [7].

Voriconazole is predominantly eliminated by hepatic metabolism. Its metabolism is limited by dose showing nonlinear pharmacokinetics due to its capacity limited elimination [8]. Plasma concentration varies between patients due to the genotype of the hepatic cytochrome P 450 (CYP) enzymes. Voriconazole is an important substrate for CYP2C19 isoenzymes which usually have frequent genetic polymorphisms. CYP2C19 genotype accounts (in addition to gender and age) for a large variability in clearance and area under the curve (AUC) of voriconazole [8,9].

Most authors suggest a steady-state serum voriconazole concentration between 1.5 and 4 μg/mL. A correlation between the drug concentration and higher serum levels of serum alkaline phosphatase, aspartate aminotransferase, and bilirubin was found [10]. 

Our case developed clinically apparent acute liver failure after treatment with voriconazole. ALT increased from 17 on day 1 to 865 on day 22 from the time voriconazole was started, following which voriconazole was discontinued. The patient developed ascites, and her mental status worsened with suspicion of hepatic encephalopathy. Her respiratory status declined, and she was in acute respiratory distress requiring high-flow oxygenation and later intubation. 

The mechanism by which voriconazole induces hepatotoxicity includes cholestatic one, which is more common, while hepatomegaly or hepatitis occurs less frequently. It rarely induces liver failure and very rarely hepatic coma [9]. Our case had a predominant hepatocellular pattern of DILI, which led to fulminant liver failure. 

Another explanation of acute liver injury in this patient could be secondary to worsening right heart failure causing congestive hepatitis. 

## Conclusions

Drug-induced liver injury may at times be catastrophic despite early discontinuation. Many offending medications may be responsible for DILI. Early recognition and discontinuation of the offending agent is the cornerstone of therapy. Although voriconazole is a safe drug, it must be remembered as one of the potential agents responsible for DILI. LiverTox is an online database of offending agents responsible for DILI.
